# Supplementary material for: Six-month pain and function outcome expectations were established for total knee arthroplasty using the smallest worthwhile effect
Source: PLoS One. 2024 Apr 30;19(4):e0300439. doi: 10.1371/journal.pone.0300439 (PMC11060529; doi:10.1371/journal.pone.0300439)
Supplement: S1 File — (DOCX) [file pone.0300439.s001.docx]

**Supplemental File 1: Standardized Script for determining smallest worthwhile effect of total knee arthroplasty**

We are trying to determine how much your knee pain and function will need to improve for you to believe that your surgery is worthwhile to you.

Total Knee Replacement

Before we begin, I would like to review with you the costs and risks of a total knee replacement. You can use this information in whatever way you choose when I ask you the remaining questions. The surgery is an inpatient procedure performed by an orthopedic surgeon. After the surgery, you will remain in the hospital for approximately 1 to 4 days. An average of six weeks of formal physical therapy is usually necessary for recovery. The 90-day perioperative costs in Savannah range from $14-17,000. Of these costs, Medicare typically covers 100% of the inpatient costs and 80% of the outpatient costs, while private insurance costs are typically higher and they vary in the amount they cover. There is a 3-5% overall complication rate. According to research, of the 90 day post-surgical potential complications, there is a 1% chance of infection, less than 1% chance for pneumonia, less than 2% chance of a blood clot, and a 1% chance of requiring a revision of your total knee. Most of these complications will result in longer hospitalization for additional treatment.

Do you have any questions about the knee replacement surgery?

Smallest Worthwhile Effect in pain for total knee replacement (using the KOOS Pain Scale)

Think about the knee pain you have, based on the pain scale you completed. Without the knee replacement surgery, you could expect your pain to continue to worsen by approximately 10-15% over the next six months. When considering knee replacement surgery, most of the improvement in pain occurs by 6 months after surgery. You scored a ___on your initial pain scale. Please complete this additional pain scale to show how much pain with activity is acceptable 6 months after surgery, considering the 10% to 15% worsening you would experience without the knee replacement surgery, in order for you to feel that the surgery was worthwhile, given the costs and risks we reviewed earlier.

Now, I am going to provide you with a scenario where I change your response to questions on the 6 month pain scale. For example, if you reported “no pain” with question # 7- “pain at night while in bed” and I shifted your response from “no pain” to “mild pain” with this activity, would you still consider the surgery worthwhile considering the associated costs and risks?

Now, let’s begin. For questions #AA and #BB on the 6 month pain scale, you reported “_________” pain with these activities. If I shifted your response from “__________” pain to “__________” pain with these activities, indicating a higher level of pain, would you still consider the surgery worthwhile considering the associated costs and risks?

#AA= the item on the 6-month KOOS-Pain scale with the lowest, least painful, item score.

#BB= the item on the 6-month KOOS-Pain scale with the second lowest, least painful, item score.

If the answer is no:

Would you still consider the surgery worthwhile if I only shifted your response on question #AA from “________” pain to “_________” pain with this activity, indicating a higher level of pain?

If the answer is yes:

Now, let us assume that you now have “_________” pain with questions #AA and BB, based on the previous scenario. If I shifted your response on two additional questions, #CC and #DD, from “_________” pain to “______” pain with these activities, indicating a higher level of pain with both activities, would you still consider the surgery worthwhile considering the associated costs and risks?

#CC= the item on the 6-month KOOS-Pain scale with the third lowest, least painful, item score.CC

#DD- the item on the 6-month KOOS-Pain scale with the fourth lowest, least painful, item score.

If the answer is no:

Would you still consider the surgery worthwhile if I only shifted your response on question #CC from “________” pain to “_________” pain with this activity, indicating a higher level of pain?

*This same procedure will continue until either the subject answers “no” to both activities or they answer “yes” to all nine items on the pain scale.

SWE in function for total knee replacement (using KOOS Function, daily living Scale)

Think about the activities which you had difficulty with on the functional scale you completed. Without the knee replacement surgery, you could expect for your functional level to continue to worsen by approximately 10-15% over the next six months. The majority of improvement in function can be expected by 6 months after surgery. You scored a ___ on the initial functional scale. Please complete this additional functional scale to show how much difficulty with activity, considering the 10% to 15% worsening you would experience without the knee replacement surgery, is acceptable in order for you to feel that the surgery was worthwhile, given the costs and risks we reviewed earlier?

I am going to provide you with a scenario where I change your response to two of the questions on the 6 month function scale. For example, if you reported “no difficulty” for question # 14- “difficulty with sitting or lying” and I shifted your response from “no difficulty” to “mild difficulty” with this activity, would you still consider the surgery worthwhile?

Now, let’s begin. For questions #AA and #BB on the 6 month functional scale, you reported “_________” pain with these activities. If I shifted your response from “__________” difficulty to “__________” difficulty with these activities, indicating a higher level of difficulty, would you still consider the surgery worthwhile considering the associated costs and risks?

#AA= the item on the 6-month KOOS-Function scale with the lowest, least difficult, item score.

#BB= the item on the 6-month KOOS-Pain scale with the second lowest, least difficult, item score.

If the answer is no:

Would you still consider the surgery worthwhile if I only shifted your response on question #AA from “________” difficulty to “_________” difficulty with this activity, indicating a higher level of difficulty?

If the answer is yes:

Now, let us assume that you now have “_________” difficulty with questions #AA and BB, based on the previous scenario. If I shifted your response on two additional questions, #CC and #DD, from “_________” difficulty to “______” difficulty with these activities, indicating a higher level of difficulty with both activities, would you still consider the surgery worthwhile considering the associated costs and risks?

#CC= the item on the 6-month KOOS-Pain scale with the third lowest, least difficulty, item score.CC

#DD- the item on the 6-month KOOS-Pain scale with the fourth lowest, least difficulty, item score.

If the answer is no:

Would you still consider the surgery worthwhile if I only shifted your response on question #CC from “________” difficulty to “_________” difficulty with this activity, indicating a higher level of difficulty?

*This same procedure will continue until either the subject answers “no” to both activities or they answer “yes” to all seventeen items on the functional scale.
