# Supplementary material for: Six-month pain and function outcome expectations were established for total knee arthroplasty using the smallest worthwhile effect
Source: PLoS One. 2024 Apr 30;19(4):e0300439. doi: 10.1371/journal.pone.0300439 (PMC11060529; doi:10.1371/journal.pone.0300439)
Supplement: S2 File — (DOCX) [file pone.0300439.s002.docx]

Supplemental File 2

Baseline comparisons between those who completed the six month follow-up and those who did not

|  | Completed follow-up (N=82)  Mean (sd) | Lost to follow-up (N=39)  Mean (sd) | P value |
| --- | --- | --- | --- |
| Sex (female) | 44 (55%) | 20 (51%) | 0.70 |
| Age (years) | 67.4(10.5) | 66.7 (8.0) | 0.80 |
| KOOS Pain | 43.8 (17.4) | 45.1 (16.0) | 0.71 |
| KOOS Function, daily activity | 45.5 (19.5) | 46.9 (18.7) | 0.71 |
| Educational level, *n* (%) |  |  | 0.29 |
| Less than high school graduate | 2 (2.5) | 1 (2.5) |  |
| High school degree | 11 (13.9) | 4 (10.0) |  |
| Some college | 20 (25.3) | 6 (15.0) |  |
| College degree | 21 (26.6) | 14 (35.0) |  |
| Some graduate school | 3 (3.8) | 2 (5.0) |  |
| Graduate degree | 22 (27.8) | 13 (32.5) |  |
| Yearly household income level in thousands of US dollars, *n* (%) |  |  | 0.59 |
| <$10k | 1 (1.3) | 2 (5.1) |  |
| $10k to <25k | 6 (7.9) | 0 (0) |  |
| $25k to <50k | 17 (22.4) | 5 (12.8) |  |
| $50k to 100k | 28 (31.6) | 22 (56.4) |  |
| >$100k | 24 (31.6) | 10 (25.6) |  |
|  |  |  |  |
|  |  |  |  |
