# Supplementary material for: Six-month pain and function outcome expectations were established for total knee arthroplasty using the smallest worthwhile effect
Source: PLoS One. 2024 Apr 30;19(4):e0300439. doi: 10.1371/journal.pone.0300439 (PMC11060529; doi:10.1371/journal.pone.0300439)
Supplement: Attachment — Submitted filename: Responses to review team.docx [file pone.0300439.s005.docx]

Reviewers' comments:

Reviewer's Responses to Questions

We are grateful to the review team for the thorough review of our paper. In response to the comments, we have highlighted the changes in red.

Comments to the Author

1. Is the manuscript technically sound, and do the data support the conclusions?

The manuscript must describe a technically sound piece of scientific research with data that supports the conclusions. Experiments must have been conducted rigorously, with appropriate controls, replication, and sample sizes. The conclusions must be drawn appropriately based on the data presented.

Reviewer #1: Yes

Reviewer #2: Yes

2. Has the statistical analysis been performed appropriately and rigorously?

Reviewer #1: Yes

Reviewer #2: Yes

3. Have the authors made all data underlying the findings in their manuscript fully available?

The PLOS Data policy requires authors to make all data underlying the findings described in their manuscript fully available without restriction, with rare exception (please refer to the Data Availability Statement in the manuscript PDF file). The data should be provided as part of the manuscript or its supporting information, or deposited to a public repository. For example, in addition to summary statistics, the data points behind means, medians and variance measures should be available. If there are restrictions on publicly sharing data—e.g. participant privacy or use of data from a third party—those must be specified.

Reviewer #1: No

Reviewer #2: No

4. Is the manuscript presented in an intelligible fashion and written in standard English?

PLOS ONE does not copyedit accepted manuscripts, so the language in submitted articles must be clear, correct, and unambiguous. Any typographical or grammatical errors should be corrected at revision, so please note any specific errors here.

Reviewer #1: Yes

Reviewer #2: Yes

Author responses to general questions: We very much appreciate the helpful feedback and guidance from the review team. We have addressed each comment below and have made what we believe to be substantive responses to each query by highlighting the changes in red.

5. Review Comments to the Author

Please use the space provided to explain your answers to the questions above. You may also include additional comments for the author, including concerns about dual publication, research ethics, or publication ethics. (Please upload your review as an attachment if it exceeds 20,000 characters)

Reviewer #1: I will specify :

1) results are not well reported in a table(supplemental file deals only with baseline findings)

Author response: Thank you for the suggestion. We have added a Table 2 to succinctly summarize the key findings in a Table.

Changes to manuscript: We have added a Table 2 with key findings reported in Table format. Please see page 10, lines 230-239.

2)line97: misses the number of participants before exclusions and denials

Author response: Thank you. The total sample screened and excluded was already fully described a few lines below this.

Changes to manuscript: No changes made.

3)line98: there is no reference to baseline daily activity level and pain at start of observation or any link to the table.

Author response: The baseline KOOS pain and Function, daily activity scores are reported in the first few lines of the Results section as well as the original Table 1 and we believe that this is the appropriate place to report this information.

Changes to manuscript: No changes made.

4)line 114: why did you prefer to use KOOS instead of other scale?This is not explained in the introduction or in the methods

Author response: Thank you for the comment. We now briefly provide a justification for the use of KOOS, one of the most commonly recommended and validated measures in osteoarthritis and arthroplasty.

Changes to manuscript: Please see lines 124-127 on page 6. “We chose the KOOS Pain and Function, daily living scales because these are two of the most commonly recommended and validated scale for patients with knee osteoarthritis and arthroplasty. Both were derived directly from the WOMAC scale and, at the time of this study, were available for public use [16,17]. “

5)line168: the sample size should be 121 while it is written 120

Author response: Thank you. The number of 120 was used in a power analysis and does not reflect the total sample recruited. The sample size calculation used an n = 120 so this is the correct number.

Changes to manuscript: No changes required.

6)in the discussion & conclusions(from line 242): you did not refer to selection bias even though your sample is mainly well educated and with good income.This could influence the understanding of the questionnaire and the aim of the study with a rejection response higher from people with a lower education who usually have worst outcome after 6 months.

Author response: Excellent point. We have added this to our limitations section as a potential source of bias.

Changes to manuscript: Please see lines 342-343 on page 15. “Additionally, our sample was generally well educated with relatively high income and this may have biased the results.”

7)line 235: the word "was" is repeated twice(TYPO)

Author response: Thank you. The typo was corrected.

Changes to manuscript: Please see line 261, page 11.

Reviewer #2: Overall Comment: This is a well-written manuscript that provides important information about patient expectations and outcomes. It is relevant to the PT and ortho community and is suitable for publication.

Please find below some minor modifications to further improve this work.

Author response: We very much appreciate your positive comment endorsing the importance of our paper.

Comment #1: The manuscript describes a technically sound method for determining 6-month post outcomes' expectations during pre-op in patients undergoing TKA. The method and the data support this conclusion.

However, I would recommend making the distinction/clarification and mentioning the "expectation" aspect of this throughout the manuscript i.e. lines 110-111, 143, 260-261, 318. The expectation is the most important nuance in this manuscript.

Author response: Thank you for this important point. We have emphasized the novelty and importance of this issue in multiple areas.

Changes to manuscript: Please see lines 118-120 on page 5 “The SWE required participants to express their expectations regarding their outcomes following TKA and this was the novel and important component in our study. “ and lines 289-90 on page 13 “ Questioning about expectations in the way we did is novel and a highly important component to our study. “

Comment #2: Introduction

- In the introduction, lines 68-79: this explains the SWE method and what it requires but also what and how it was used in this study. This should be moved to material and methods rather than the intro. Please consider adjusting accordingly.

Author response: Given the importance of the SWE method and it’s key role in our study, we actually think it is important to emphasize it in the introduction to make the reader aware of the role of SWE and expectation assessment in our study. Given the novelty of this approach we think it’s quite important to make the reader aware of this method in a brief and general way before providing more detailed description in the Methods. This section also is written in a way that provides a more general description of this new approach, an issue that we believe to be important enough that the reader should have some sense of this method before getting to the detail. We have added some elaboration to guide the reader on why we believe this aspect of the study to be important.

Changes to manuscript: Please see lines 79-84 on page 4. “By determining patient expectations in this more formal way, it is our view that surgeons, other clinicians, and patients may benefit from a more thorough determination of expectations in the context of key variables including costs, risks and benefits, relative to no TKA. Determining expectations in this way may allow for a more accurate estimation of patient outcome expectations and enhance satisfaction, an important outcome for both surgeons and patients. “

- Add a few sentences in the intro about the importance that finding the SWE could have clinically for surgeons and for patients, with references, if possible.

Author response: We appreciate the suggestion and believe this adds important information to the general description already included in the introduction and addressed in the comment above.

Changes to manuscript: This point overlaps with the point above. Please see lines 79-84 on page 4. “By determining patient expectations in this more formal way, it is our view that surgeons, other clinicians, and patients may benefit from a more thorough determination of expectations in the context of key variables including costs, risks and benefits, relative to no TKA. Determining expectations in this way may allow for a more accurate estimation of patient outcome expectations and enhance satisfaction, an important outcome for both surgeons and patients. “

Comment #3: Materials and Methods

- Lines 94-95: consider adding a subheading called Recruitment or Study Population or something like that since you are using subheadings for the rest of this section.

Author response: Thank you. A new heading has been added.

Changes to manuscript: Subheading added to Materials and Methods section on page 4.

- line 95: you mention 121 persons, better use "patients"

Author response: Thank you for the suggestion. The standard approach when describing patients recruited for a research study is to consistently describe them as “participants.” We now consistently use the word participants. The description in our paper makes clear that our participants were patients at the time of the study.

Changes to manuscript: Changed to “participants” throughout the paper when referring specifically to the participants in our study.

- line 97: "men and women", some people are non-binary etc. I would say "all adult patients aged 45-90, diagnosed with symptomatic knee osteoarthritis (OA), and scheduled for primary TKA by one of our nine participating surgeons were considered for inclusion in this study regardless of race or gender."

Author response: Thank you. We have made this change.

Changes to manuscript: Please see line103-104 on page 5. “All adult patients aged 45-90 years,…”

- line 95, you mention 121 persons. then say 10 were excluded (line 101), then say 129 met criteria but declined (line 102) and you ended up with 121 patients. While I understand this, it would be nicer and easier to the reader to see the breakdown of those number. Something like the following:

"This was a secondary analysis of a prospective longitudinal study patients scheduled for a unilateral TKA for osteoarthritis at a hospital in Savannah, Georgia, USA. All adult patients aged 45-90, diagnosed with symptomatic knee osteoarthritis (OA), and scheduled for primary TKA by one of our nine participating surgeons were considered for inclusion in this study regardless of race or gender. Participants were excluded if they were scheduled for revision TKA, simultaneous bilateral knee arthroplasty, unicompartmental knee arthroplasty, or knee arthroplasty 100 for cancer or rheumatic disease. A total of 10 patients were excluded because they met one or more exclusion criteria. A total of 250 met inclusion criteria: 129 declined participation after and 121 provided written informed consent and were included in this study."

Author response: The suggestion is very much appreciated and incorporated into the paper.

Changes to manuscript: Please see lines 102-110 on page 5. “This was a secondary analysis of a prospective longitudinal study patients scheduled for a unilateral TKA for osteoarthritis at a hospital in Savannah, Georgia, USA. All adult patients aged 45-90 years, diagnosed with symptomatic knee osteoarthritis (OA), and scheduled for primary TKA by one of nine participating surgeons were considered for inclusion, regardless of race or gender. Participants were excluded if they were scheduled for revision TKA, simultaneous bilateral knee arthroplasty, unicompartmental knee arthroplasty, or knee arthroplasty for cancer or rheumatic disease. A total of 10 patients were excluded because they met one or more exclusion criteria. A total of 250 met inclusion criteria: 129 declined participation after and 121 provided written informed consent and were included in this study.”

- Apart from baseline characteristics, it is important to add the chief complaints of patients/the reason for TKA. As this alone might alter the expectation, pain and outcomes...

Author response: This is an excellent suggestion though these data were not collected beyond completion of the KOOS scales so we are unable to provide this information. With this said, it seems clear that complaints of pain with activity and compromised function are likely subsumed into the KOOS scale ratings. We have noted this in our limitations section.

Changes to manuscript: Please see lines 342-346 on page 14. “We did not specifically determine participants’ chief complaints but it is likely that the great majority sought TKA because of knee pain and compromised function, and both are captured by KOOS scale scores [48]. “

- line 108: would omit Virgina Commonwealth University. Replace by "the university Institutional Review Boards".

Author response: There is only one university IRB and we would prefer to keep the name of the institution in case there is any question of who approved the review.

Changes to manuscript: No changes made.

- line 118: please if possible cite a source for the "10% to 15% average worsening expected without TKA" as this is a big claim.

Author response: Thank you. We have provided two references to support this estimate.

Changes to manuscript: Please see lines 130-131 on page 6. “…10% to 15% average worsening expected without TKA, and given the costs and risks of surgery [18,19].”

- line 124-127: excellent explanation

Author response: thank you!

- line 146-147: annual income and educational level are mentioned. Consider explaining why these were collected/if looking at SES, etc.

Author response: They were included to provide a thorough description of sample characteristics. This point has been added to the paper.

Changes to manuscript: Please see line 157 on page 7. “To thoroughly describe our sample,…”

- line 162: regarding the PASS, it remains unclear to the reader, without going back to the reference, whether the PASS only consists of one question (lines 163-165) or it it's a full questionnaire (like mentioned in line 163). Please clarify.

Author response: We have added a brief statement to clarify that this was a single item.

Changes to manuscript: Please see line 175 on page 8. “The single item PASS question was…”

Comment #4: Data analysis

- line 167: why was the study powered to detect a 50th percentile estimate only. Why not 75th as per the KOOS measure in other publications. Please justify/clarify.

Author response: We wanted to reasonably concise estimate for the average patient. This rationale was added to the paper.

Changes to manuscript:

- line 169-171: why was the study not powered?

Author response: The study was powered for baseline scores but not for follow-up scores because we were unclear about the rate of follow-up and the baseline scores were considered the most critical for SWE estimation.

Changes to manuscript: Please see lines 179-184 on page 8. “ Our study was originally powered to detect a 50th percentile estimate for the SWE. A sample size of 120 would result in a 95% confidence interval of 0.40 to 0.60 which we believed to be reasonable for estimating the 50th percentile for the SWE for the average patient. The study was not powered to establish precision for the six-month postoperative KOOS measures reported on in the current study because it was the baseline scores that were most critical for SWE estimation.”

- lines 177-182: the correct tests were chosen and the statistical analyses serve the purpose of this study.

Author response: Thank you.

Comment #5: Results

- For clarity purposes, consider using subheadings for the results either as per the materials and methods or as per the outcomes.

Author response: Thank you – subheadings have been added to the Results section.

Changes to manuscript: Please see new subheadings beginning on page 9.

- Line 188: specify that it's the KOOS questionnaires.

Author response: Thank you. This has been added.

Changes to manuscript: Please see line 203 on page 9. “participants completed six-month follow-up KOOS questionnaires…”

- Line 195: good justification, please provide the data as supplemental file #3.

Author response: Yes, we have added the raw data as a supplemental file.

Changes to manuscript: Please see line 211 on page 9. “Raw data are provided in Supplemental file 3.”

- line 236-237: weird sentence, please review.

Author response: Thank you. We have revised this sentence to make it clearer.

Changes to manuscript: Please see line 261-265 on page 11-12. “Similarly, the Pearson chi square test for KOOS Function was also significant with Χ2 (1, N = 82) = 23.479, p < 0.001. A total of 50% (4/8) of participants whose actual six-month KOOS scores were less than their final expected KOOS Function, daily activity scores were dissatisfied with their outcome. A total of 2.7% (2/73) of participants who scored equal to or better than their final expected KOOS Pain score were dissatisfied with their outcome.”

Comment #6: Discussion

- line 245: define KA as it has not been used before in the manuscript, or use TKA which has. Adjust manuscript accordingly.

Author response: Our apologies for the typo. This should be TKA. This was corrected throughout the paper.

Changes to manuscript: TKA used throughout the paper.

- line 250-254: excellent and relevant

Author response: Thank you!

- line 256/269: it would be interesting to talk about the underestimation of estimates too. I might have misunderstood but it's also unclear how overestimation lead to better satisfaction (line 269) rather than lesser. It would be nice to discuss all three outcomes, underestimation, adequate estimation and overestimation.

Author response: Thank you for the suggestion. We see that the meeting and exceeding expectations as one group but with this said, we have addressed this comment by adding material to the discussion.

Changes to manuscript: Please see lines 284-287 on page 12. “Patients who fall short of baseline expectations at the 6-month postoperative time point were significantly more likely to be dissatisfied with their outcome (i.e., approximately 50% dissatisfaction rate versus an estimated 2% dissatisfaction rate for those meeting or exceeding expectations).”

- line 274: define SDM

Author response: Thank you. We have defined the acronym.

Changes to manuscript: Please see line 304 on page 13. “During the shared decision making (SDM) discussion..”

Comment #7: Conclusion

- Adequate, answers the research question and provides solutions.

Author response: Thank you!

Well done overall! Very interesting.

Author response: Very much appreciated.
